# The oil-dispersion bath in anthroposophic medicine – an integrative review

**DOI:** 10.1186/1472-6882-8-61

**Published:** 2008-12-04

**Authors:** Arndt Büssing, Dirk Cysarz, Friedrich Edelhäuser, Gudrun Bornhöft, Peter F Matthiessen, Thomas Ostermann

**Affiliations:** 1 Medical Theory and Complementary Medicine, University of Witten/Herdecke, Gerhard-Kienle-Weg 4, 58239 Herdecke, Germany; 2 Integrated Studies of Anthroposophical Medicine, University of Witten/Herdecke, Gerhard-Kienle-Weg 4, 58239 Herdecke, Germany

## Abstract

**Background:**

Anthroposophic medicine offers a variety of treatments, among others the oil-dispersion bath, developed in the 1930s by Werner Junge. Based on the phenomenon that oil and water do not mix and on recommendations of Rudolf Steiner, Junge developed a vortex mechanism which churns water and essential oils into a fine mist. The oil-covered droplets empty into a tub, where the patient immerses for 15–30 minutes. We review the current literature on oil-dispersion baths.

**Methods:**

The following databases were searched: Medline, Pubmed, Embase, AMED and CAMbase. The search terms were 'oil-dispersion bath' and 'oil bath', and their translations in German and French. An Internet search was also performed using Google Scholar, adding the search terms 'study' and 'case report' to the search terms above. Finally, we asked several experts for gray literature not listed in the above-mentioned databases. We included only articles which met the criterion of a clinical study or case report, and excluded theoretical contributions.

**Results:**

Among several articles found in books, journals and other publications, we identified 1 prospective clinical study, 3 experimental studies (enrolling healthy individuals), 5 case reports, and 3 field-reports. In almost all cases, the studies described beneficial effects – although the methodological quality of most studies was weak. Main indications were internal/metabolic diseases and psychiatric/neurological disorders.

**Conclusion:**

Beyond the obvious beneficial effects of warm bathes on the subjective well-being, it remains to be clarified what the unique contribution of the distinct essential oils dispersed in the water can be. There is a lack of clinical studies exploring the efficacy of oil-dispersion baths. Such studies are recommended for the future.

## Background

Complementary and alternative medicine (CAM) has become increasingly popular over the last decades. Several surveys suggest an increasing demand and use of CAM in almost all western nations[1]. While in these surveys CAM was reported to be mainly used for common diseases like allergic reactions and common cold, we have to state a much broader variety of diseases covered by an expanding spectrum of CAM therapies.

One of the whole medical systems of CAM, which is quite popular in Germany, is the Anthroposophic Medicine (AM), introduced by Rudolf Steiner in the beginning of the 20^th ^century [2]. Based on a comprehensive view of the human body, health in AM conceptually depends on a harmonious relationship and interaction between the physical body, vital force, soul, and ego [3]. Therefore, therapies of AM may address each of these four entities to support the reestablishment of their harmonious interrelations (e.g., to enhance the salutogenetic capacities of the patients, or to strengthen their autonomy [4]).

Over the time, AM has developed a variety of pharmacological and non-pharmacological therapies. With respect to pharmacological therapies of AM, interventions like mistletoe therapy for cancer treatment [5-7] or Cardiodoron, a composition of extracts of blossoms from *Primula officinalis *and *Onopordon acanthium *and from the herbs of *Hyoscyamus niger *for cardio-respiratory coordination [8,9], have been subject of several investigations from both basic research to systematic review or meta-analysis.

Among elderly patients with chronic diseases, 2% use AM applied by medical doctors, and at least 4% of patients with cancer [10] – although more 50–70% of cancer patients may use plant extracts from *Viscum album *which are part of the AM concept to treat cancer patients. Thus, these 2–4% stated above probably represent a lower bound of the factual usage by the population.

In addition, AM offers a variety of non-pharmacological therapies, but only few such therapies have been investigated extensively. It has been shown that Anthroposophic Speech Therapy has an impact on cardiovascular oscillations (e.g., cardiorespiratory synchronization during hexameter recitation [11]), and may have beneficial effects on the cardiorespiratory interaction [12]. Whether these effects may have a 'cardio-preventive effect' or not, remains to be clarified. On the other hand it has been shown that slow breathing improves baroreflex sensitivity and decreases blood pressure in essential hypertension [13]. Because speech therapy employs similar breathing patterns with low breathing frequencies, such effects are supposable. Other non-pharmacological therapies such as Rhythmic Embrocation showed an improvement of pain perception and mood in patients with chronic pain [14], while Rhythmic Massage resulted in a long-term reduction of disease symptoms and quality of life in patients with chronic diseases [15] A recent systematic review investigated the clinical effects of Anthroposophic Eurythmy Therapy [16] and stated positive treatment effects with clinically relevant effect sizes in most cases.

**Figure 1:**
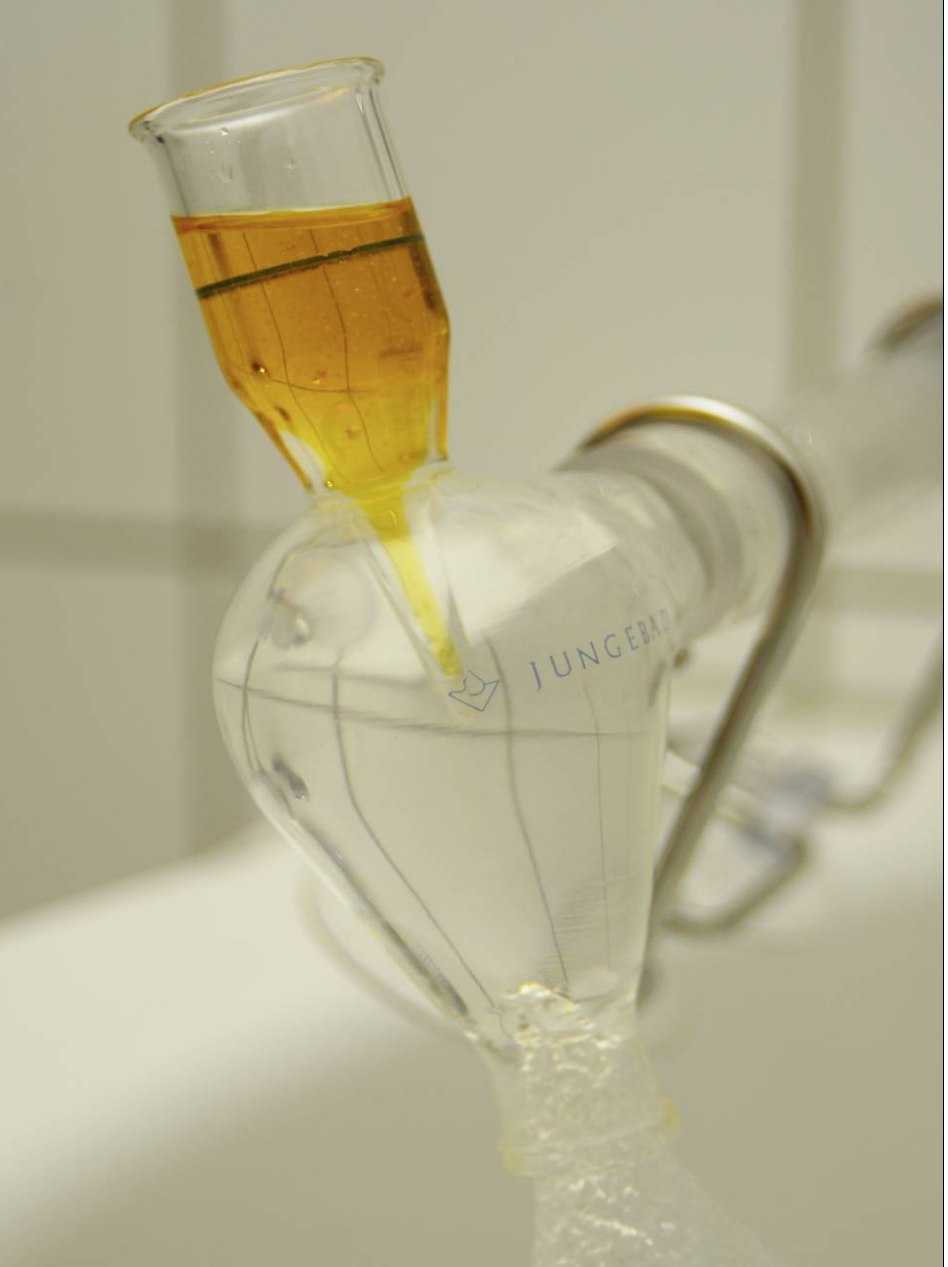
**Vortex mechanism of the oil-dispersion bath**. With kind permission of Florian Junge, Bad BOll

In the context of balneology, AM offers a unique contribution, i.e., the Oil Dispersion Bath (ODB). In 1920, Rudolf Steiner, the founder of Anthroposophy suggested the use of finely-dispersed oil in water in [17]. Analogous to the process of oil synthesis in plants and fruits (e.g. olives, 'oil forming process'), he suggested to enable the patients by activating his salutogenetic capacities of the organism with water as a mediator for the essential oils. According to Steiner, if a patient's 'warmth organism' is affected or weakened, one may connect it to the 'warmth processes' of nature, i.e., the 'oil forming process' of flowering plants, and thus introduce finely dispersed oil into a bath to strengthen the weakened 'warmth organism' of the patient [17]. According to AM, the oils are related to the ethereal 'flowering process' of plants (in terms of a formation or accumulation of 'warmth') and address particularly the 'self organization' which in turn has an influence on its 'warmth organism'. Based on these recommendations, in the 1930s, the massage therapist and medical bath attendant Werner Junge began to develop a system for very fine oil dispersal in water and developed a vortex mechanism which churns water together with essential oils into a fine mist [18]. The oil-covered water droplets empty into a 35 to 37°C (95 to 99°F) warm water tub, where the patient immerses for 15–20 minutes (Fig. 1).

Main indications are the regulation of the 'warmth organism', particularly in patients with arthrosis, gout, and arteriosclerosis [19], but also neurological conditions such as polyneuropathia [20], to improve hypotonic blood pressure [21], and improvement of the 'structuring metabolization' in terms of a tendency to deformation [21,19]. Patients with cardiovascular diseases or neurological diseases may use slightly colder bathes, i.e., 32 to 34°C (90 to 93°F).

The assortment of the oils, according to the criteria of AM are as follows: Oils from roots are used to strengthen the nervous system; oils from flowers are applied to enhance the capacities of the metabolic system; oils from seeds are supposed to impact the heart and oils from fruits are recommended for improving vascular circulation.

After the bath, the patients should be covered in dry and warm clothes and blankets for at least half an hour in a quiet atmosphere to let the applied warmth saturate the body [19].

Although experts often recommend ODB and despite of its frequent usage in the treatment of in-patients in anthroposophic hospitals (in most cases ODB are applied daily and later three times per week) together with other therapies of AM, still less is known about the physiological and clinical effects. We pragmatically intended to overview the current literature on this unique therapy in a systematic review.

## Methods

We intended to search for English, French or German language case reports, studies and controlled trials which address specific treatment effects of ODB in a clinical setting without any restrictions to indications or outcome measures. Comments, opinions, and theoretical considerations were excluded. To get a first overview, the following databases were used: Medline, PubMed, Embase, AMED and CAMbase. The search terms were "oil dispersion bath", "oil bath" and their respective translations in German and French. Moreover, an internet search was performed using Google Scholar adding the search terms "study" and "case report" to the above search terms. Finally, we screened the literary estate of Werner Junge and asked several experts for gray literature, i.e., material not found in conventional channels such as publishers with a lack of strict bibliographic control, and thus not listed in the above mentioned databases.

All articles found this way were fully read and their reference lists were checked for further relevant publications. The complete search was performed between June and October 2007. Report identification was performed by GB, TO and AB for the clinical studies, and as an additional reviewer for the experimental studies by DC. Data extraction was primarily performed by GB, and checked by TO and AB. We analyzed the full length articles rather than just the abstracts. Disagreements were resolved by consensus.

As the studies were quite heterogeneous including a variety of perspectives, but not any randomized controlled trial, we decided to classify them with respect to indication, treatment setting, research design, the number of patients involved and the outcome measures/results given in the studies/reports. The reporting of the results adhered, if possible and appropriate at all to the MOOSE guidelines [22]. Relying on a checklist for the assessment of methodological quality of the identified studies was dispensable, because the identified studies were almost weak in methodological quality at all. Nevertheless, we intended to refer to the clear description of aim and objective, outcome measures, characteristics of included patients, follow up/drop outs, interventions, principal confounders; participants representative; blinding, randomization, multicenter, same recruitment period. Because most of these issues were not fulfilled in the identified studies, we decided to describe the main relevant flaws in the results.

## Results

After excluding theoretical work, descriptive literature and brochures and leaflets of pharmaceutical industries with limited scientific background, we identified 12 relevant articles. Among them, 1 prospective clinical study, 3 experimental studies (enrolling healthy individuals), 5 case reports, and 3 field-reports. The methodological quality of most studies was weak (i.e., presentation of inconclusive results, limited description of patients' characteristics, unclear process of patient selection, unclear inclusion-/exclusion criteria, unclear therapeutic aims, small number of enrolled patients, no control group, etc.). Main indications were internal/metabolic diseases and psychiatric/neurological disorders. Table 1 gives a detailed overview on indications, setting, study type, and outcome parameters.

**Table 1:** Design of clinical studies on the effects of ODB and outcome parameter

**Authors**	**Type of study, N**	**Indications**	**ODB Application**	**Outcome**
**Experimental studies**				
*Nienhaber *[23]	physiological experiment (n = 8)	Healthy subjects	Water ODB, Rosmary-Kneipp-Bath, Rosmary-ODB, Whirl Bath and Relaxation (Baths about 30 min, thermoindifferent)	Orthostatic Reactions of Pulse and Respiration: standing pulse frequency decreased most after ODB and diastolic RR-increasing strongly subdued
*Römmelt *[24]	pharmacological experiment (n = 6)	Healthy subjects	30 min Pinus oil ODB	Percutaneous resorption of terpenes after ODB
*Römmelt *[25]	physiological experiment (n = x)	Healthy subjects	20 min ODB with Rosmary Oil, Pinus Oil and hay flower oil	Percutaneous resorption of terpenes after ODB

**Clinical studies**				
*Braunstein *[26]	Prospective study (n = 17)	Schizophrenic (n = 9) and depressive patients (n = 8)	Series of 10 Thyme-ODB	relaxation, schizophrenic patients showed immediate and long term improvement of well-being, depressive patients stagnated in well-being after 5 baths

**Case reports**				
*Roknic et al*. [32]	Case reports (n = 7)	Schizophrenic disorders (n = 3); purulent bronchitis/asthma bronchiale; hyperkinesias; Down-Syndrome related depression; Hospitalism	ODB with different oils among other therapeutic interventions	general improvements
*Liebing *[33]	Case reports (n = 3)	Hysteria; attention deficit hyperactive disorder; autism	Series of ODBs (with Stibium sulfuratum, *Hypericum*, *Prunus spinosa*, respectively	General improvement and feeling of inner warmth; developmental improvement (movements, balance, sense of touch; developmental improvement (joy for life and willingness to learn)
*von Rottenburg *[31]	Case report (n = 1)	Neurodermatitis of a 4-year old girl		After 12 ODBs, significant improvement of skin lesions; marginal relapses, which diminished in their intensity; finally total regeneration of skin affections
*Phethean *[34]	Case report (n = 1)	Richard: 9 years old developmental delay	ODB with different oils	Richard: Strengthening, inner resolution, more peaceful
*Lavington *[35]	Case report (n = 1)	Angela: 12 years old, Autism	ODB with different oils	Angela: relaxation, inner calming

**Field reports**				
*Alles *[29]	Field report	Diabetes mellitus Type I & II	Rosmary ODB: 5 × weekly within 4 weeks and 3 × weekly within 8 weeks	No results presented; Type 2 diabetes (insulin-dependent) was claimed to be cured; in contrast, no improvements of metabolic situation in patients with type 1 diabetes
*Deckers *[30]	Field report	Rheumatic Diseases	Various bathes described, i.e. saline bath, sulfur bath, and ODB and stinging-nettle bath	No results presented; just a statement that complaints and subjective well-being improves
*Rimpau *[20,28]	Field report	Painful Neuropathy	10-day-in patient treatment with daily Rosmary-ODB	No results presented; Subjective improvements

### Physiological and pharmacological effects

Nienhaber [23] investigated the effects of Water Bath, Rosmary-Kneipp-Bath, Rosmary-ODB, Whirl Bath and thermo-indifferent Relaxation Baths (about 30 min) on cardiovascular regulation of eight healthy subjects. After Rosmary-ODB, the orthostatic test showed the largest decrease of standing pulse rate and a strongly subdued increase of diastolic blood pressure. This indicated an improved regulatory capacity in terms of a stabilization after the orthostatic challenge.

In two experimental studies, Römmelt [24,25] examined the concentration of terpenes from ODB etheric oils in the whole blood of healthy individuals. After 30 minutes ODB, he found a significant increase of the terpene concentration in the blood, indicating its rapid transdermal resorption. Römmelt [24] stated, that the yielded blood concentrations could be of pharmacological relevance.

### Clinical effects

The clinical study of Braunstein [26] investigated nine schizophrenic and eight depressive patients who were treated with a series of Thyme-ODB. Main outcome parameters were the vagotonic response as an indicator of physical relaxation and mood as assessed by the patient itself and therapist using a 5-item visual analogue scale according to Aitken, and Zerssens's Bf-S mood inventory. All patients showed a vagotonic shift towards relaxation as indicated by a general decrease of pulse rate and a change of blood pressure according to the initial blood pressure: in patients with elevated blood pressure the blood pressure decreased, whereas patients with normal blood pressure did not change. This effect could be the result of a normalization or regression to the mean, respectively, as suggested for similar effects [27]. Nevertheless, these effects occurred during the ODB and remained also during rest after the bath. While schizophrenic patients showed a significant improvement of well being during time, depressive patients reached maximum improvements of well-being after 5 baths with less inter-rater agreement. However, both the pharmacological and psychotherapeutical treatment of the patients remained unchanged during the course of the study.

The neurologist Rimpau [20,28] published a field report and stated that ODB was an essential part of therapeutic spectrum of his clinic; it was applied to patients with polyneuropathy, sleep disorders, Parkinson's disease, lumbo-ischialgia, and spastic pareses. He aimed to investigate putative easing effects of ODB on painful polyneuropathy, and experimented with different oils. According to his experience, in-patients receiving 3–5 Rosmary-ODB per week (during a 4–6 week period) may benefit from this intervention. During the first bathes some patients reported a dys-/paresthesia, which improved spontaneously, while some patients showed allergic skin reactions towards the ingredients and had to stop the ODB. All together, among 500 in-patients and 1,000 out-patients, it was stated that about 50 patients with painful polyneuropathy may benefit from the treatment. Unfortunately, no relevant data to underline this suggestion were presented. Moreover, the author was unable to enroll an adequately large sample of patients to conduct a clinical study.

Alles [29] published preliminary results of the ODB effects on patients with diabetes mellitus. This indication is based on theoretical considerations of Rudolf Steiner. Alles stated that according to his experiences, Rosmary-ODB 5× per week within one months and 3× per week for two month may heal patients with type 2 diabetes (non-insulin dependent). In contrast, patients with type 1 diabetes had no improvement of their metabolic situation. However, the results of the mentioned multi-center pilot study were not published.

Although another field study enrolling patients with chronic polyarthritis mentioned a relaxation of patients in the water [30], there were no specific results presented.

The results of the field reports were affirmed by some case reports which were of good descriptive quality in the practical context of the therapists rather than of scientists

Rottenburg [31] showed that ODB was effective in a 4-year old girl suffering on neurodermatitis. Parallel to an improvement in well-being and symptom-relief after a series of ODBs, relapses were reported which diminished in their intensity up to a total regeneration with no more neurodermatitis episodes in the follow up.

Roknic et al. [32] reported about 7 patients with internal or psychiatric/neurological disorders who were treated with ODB among other therapeutic interventions. The authors described general improvements in terms of feelings of inner warmth, openness to future, inner stability, deepened body awareness etc.

Liebing [33] reported on three patients with either hysteria, attention deficit hyperactive disorder or autism, receiving series of ODBs (with Stibium sulfuratum, *Hypericum*, and *Prunus spinosa*, respectively). She reported a general improvement, feelings of inner warmth, and developmental improvement (i.e., movements, balance, sense of touch; and joy for life and willingness to learn).

Phethean [34] described the case of a 9-year old boy with developmental delay. After a series of ODB he found him more peaceful, with higher strengthening and inner resolution.

Lavington [35] treated a 12-year old autistic girl and reported a relaxation and inner calming.

## Discussion

This article aims at overviewing the existing literature on the effects of ODB. We found a small number of clinical and field studies which mainly focused on the exploration of physiological effects of ODB. The studies can roughly be subdivided into two groups: (1) mechanisms of transdermal resorption of the oil, and (2) regulatory effects of ODB. While there are good clinical studies which confirm that conventional balneotherapy is an effective treatment modality in elderly patients with osteoarthritis of the knee or with chronic low back pain [36], or can alleviate low back pain [37], there is an obvious lack of clinical studies on ODB which address the unique contribution of the oil dispersal in water.

Several effects could be depending on the medium used. Balogh et al. [37] found that bathing in mineral water resulted in a statistically significant improvement of pain, mitigation of muscle spasm, alleviation of local tenderness, enhanced flexion-extension and rotation of the spine, which persisted as long as 3 months after the completion of balneotherapy. In contrast, hydrotherapy with tap water resulted only in the temporary improvement of just a single parameter: the visual analogue scale score improved significantly.

With respect to the usage of distinct oils dispersed in water, one may refer to findings of Römmelt [24,25], who found in healthy individuals an increased absorption of terpenes from bath additives containing pine needle oils or rosmary oil. Terpenoids are a subclass of the prenyllipids (i.e., terpenes, prenylquinones, and sterols) and represent the oldest group of small molecular products synthesized by plants. The major components of rosemary oil include monoterpene hydrocarbons (alpha and beta-pinene), camphene, limonene, camphor, borneol, cineole, linalool, and verbinol. Rosemary oil is known to possess marked antibacterial, antifungal, and antiviral properties; moreover, its exerts significant antioxidant activities depending on the terpenes [38-40]. Nevertheless, the clinical significance of a higher terpene concentration in the blood of patients or healthy volunteers using ODBs still remains unclear and has to be investigated in clinical studies.

The cardiovascular responses during water immersion show similar effects compared to ODB: a decrease of heart rate and blood pressure [41]. Hence, although the cardiovascular responses during and after ODB indicate beneficial effects, it remains to be explored if the dispersed oil has specific effects on cardiovascular regulation.

## Conclusion

Beyond the obvious beneficial effects of warm baths and the therapist's attention on the subjective well-being, it remains to be clarified what the unique contribution of the distinct essential oils dispersed in the water can be. In addition to further basic work that explores how the oil dispersion bath may influence physiological parameters beyond the effects of warm bath alone, well designed controlled clinical studies exploring the efficacy of ODB are strongly recommended to create a profound evidence base for this unique form of treatment.

## Competing interests

The Chair of Medical Theory and Complementary Medicine received financial support with a grant of the Raphael Medical Centre, Hildenborough, Kent, GB.

## Authors' contributions

AB, GB, TO and DC analyzed and reviewed the data, AB, TO and DC drafted the manuscript. PFM and FE contributed to draft the manuscript. AB and TO were principle authors of the paper, and had full access to the data. All authors contributed to manuscript revision and approved the final manuscript.

## Pre-publication history

The pre-publication history for this paper can be accessed here:


